# Author Correction: Parallels between experimental and natural evolution of legume symbionts

**DOI:** 10.1038/s41467-018-07205-x

**Published:** 2018-11-02

**Authors:** Camille Clerissi, Marie Touchon, Delphine Capela, Mingxing Tang, Stéphane Cruveiller, Clémence Genthon, Céline Lopez-Roques, Matthew A. Parker, Lionel Moulin, Catherine Masson-Boivin, Eduardo P. C. Rocha

**Affiliations:** 1 Microbial Evolutionary Genomics, Institut Pasteur, 28 Rue Dr. Roux, 75015 Paris, France; 2 UMR3525, CNRS, 28 Rue Dr. Roux, 75015 Paris, France; 3 LIPM, Université de Toulouse, INRA, CNRS, 31326 Castanet-Tolosan, France; 4 LABGeM, Génomique Métabolique, Genoscope, Institut François Jacob, CEA, CNRS, Université d’Evry, Université Paris-Saclay, 2 Rue Gaston Crémieux, 91057 Evry, France; 5 INRA, US 1426, GeT-PlaGe, Genotoul, 31326 Castanet-Tolosan, France; 6 Department of Biological Sciences, State University of New York, 4400 Vestal Parkway East, PO Box 6000, Binghamton, NY 13902 USA; 7 IRD, Cirad, Université de Montpellier, IPME, 911, Avenue Agropolis—BP64501, 34394 Montpellier Cedex 5, France

Correction to: *Nature Communications* 10.1038/s41467-018-04778-5, published online 11 June 2018

Clémence Genthon and Céline Lopez-Roques, who performed sequencing, were inadvertently omitted from the author list. This has now been corrected in the PDF and HTML versions of the Article.

