# Supplementary material for: Parallels between experimental and natural evolution of legume symbionts
Source: Nat Commun. 2018 Jun 11;9:2264. doi: 10.1038/s41467-018-04778-5 (PMC5995829; doi:10.1038/s41467-018-04778-5)
Supplement: Supplementary file 1 — Supplementary Information [file 41467_2018_4778_MOESM1_ESM.pdf]

Supplementary Information

Parallels between experimental and natural evolution of legume symbionts

Clerissi *et al.*

Supplementary Note 1 2

Supplementary Figures 4

Supplementary Tables 18

Supplementary References 28

Supplementary Note 1

Population genomics of *C. taiwanensis*

To study the population structure of *C. taiwanensis*, we sequenced or collected from public databases the genomes of 43 strains, plus 15 from other *Cupriavidus* spp., and 31 from *Ralstonia* spp. The core genome phylogeny of the two genera was rooted between them (Supplementary Fig. 13), in accordance with the literature [1], and was well resolved (Supplementary Fig. 2A and Supplementary Data 2). It allowed the definition of subclades with increasing phylogenetic span, from *C. taiwanensis* (Ct) and *R. solanacearum* (Rs) to the respective complete genera (Cg and Rg, details in Supplementary Fig. 2). The average nucleotide identity (ANIb) between *C. taiwanensis* genomes was often lower than the proposed cutoff for the definition of bacterial species (94%-96%) [2] (Supplementary Data 4), and its analysis in the context of the phylogeny suggests the presence of around five different species (Fig. 2, Supplementary Figs 2 and 4). The distribution of strains within these putative species was not significantly associated with the plant species from which the bacteria were isolated. Further work will be needed to detail the specificities of the different putative species within the complex.

C. taiwanensis genomes are among the smallest in its genus (~6.8 Mb, Supplementary Fig. 2D, Supplementary Data 3 for all genewise information). Even if the transition towards symbiosis occurred because of the acquisition of the large pRalta plasmid, the genomes of the *C. taiwanensis* complex seem thus to have endured significant gene loss. Similar genome reduction processes have been identified in the evolution of symbiosis of different bacterial models [3], but might not be expected in this bacterium that is thought to be originally a free-living and is now a complex of facultative mutualistic species.

The core genome of *C. taiwanensis* contains half of the genome (Supplementary Fig. 2E), and shows particularly high rates of nucleotide diversity among genes encoding regulatory proteins, plasmid transfer, and the tight-adhesion pilus (hereafter named Tad) (Supplementary Data 9). Core gene families showed extensive evidence of recombination (47% failed the PHI test), and recombination added almost as much polymorphism as mutation (43% of the total, according to ClonalFrameML). We identified fewer recombination events than expected in the symbiotic genes (relative to the rest of the core genome), in contrast to previous works in rhizobia from α-Proteobacteria [4–6]. This may be a consequence of the biogeographical distribution of the different lineages (recombination being hard to detect between very closely related taxa), or the loss of the plasmid conjugative apparatus in certain strains.

The pan genome was large (3.4 times the size of the average genome), and rarefaction curves suggest that further sampling will still lead to the identification of novel genes (Supplementary Fig. 2E). It includes a large number (19,316) of accessory gene families that are lacking in the core genome. In spite of the observed large variability of gene repertoires, the gene repertoire relatedness (GRR) between closely related genomes, computed as the ratio between the number of orthologs and the number of genes of the smallest genome, is highly correlated with the ANIb values (Supplementary Fig. 2F). Hence, *C. taiwanensis* is a genetically diverse complex of lineages that are diverse enough to become candidates to novel species. This diversity was partly the result of numerous events of recombination and horizontal gene transfer.

Comparative population genomics of *R. solanacearum* and *C. taiwanensis*.

The experimental evolution of rhizobia was done on *R. solanacearum* GMI1000, whereas the natural process took place on the ancestor of *C. taiwanensis*. To compare the two processes one needs to previously identify the differences and similarities between the two species in terms of population genomics. The *R. solanacearum* clade was separated in the three groups previously proposed to be different species [7]. The core genome of *R. solanacearum* corresponds to 51 % of the size of the average proteome (~4815 genes, Supplementary Data 2). Hence, the proportion of core genes is comparable between the two species, even if *C. taiwanensis* genomes have more genes. *R. solanacearum* showed lower rates of recombination, since ClonalFrameML inferred that mutations generated 2.25 times more polymorphism than recombination in *R. solanacearum*. *R. solanacearum* GMI1000 and *C. taiwanensis* LMG19424 have different repertoires of accessory genes (relative to the pan genomes of their respective species). In particular, the most specific functional feature of GMI1000, relative to *C. taiwanensis*, was the abundance of extracellular proteins (T3SS effectors, Supplementary Data 11).

The intersection of the core genomes of *C. taiwanensis* and *R. solanacearum* (1431 genes) allows the direct comparison of the diversification patterns of both species. The nucleotide diversity of the orthologs in the two species in this supra-core genome was significantly correlated (Spearman's rho = 0.57, $p < 0.0001$). In particular, genes of the Tad system were highly diverse in both species (Supplementary Data 9 and 11). Overall, *R. solanacearum* and *C. taiwanensis*, have different gene repertoires, but there are similarities in their rates of diversification.

Supplementary Figures

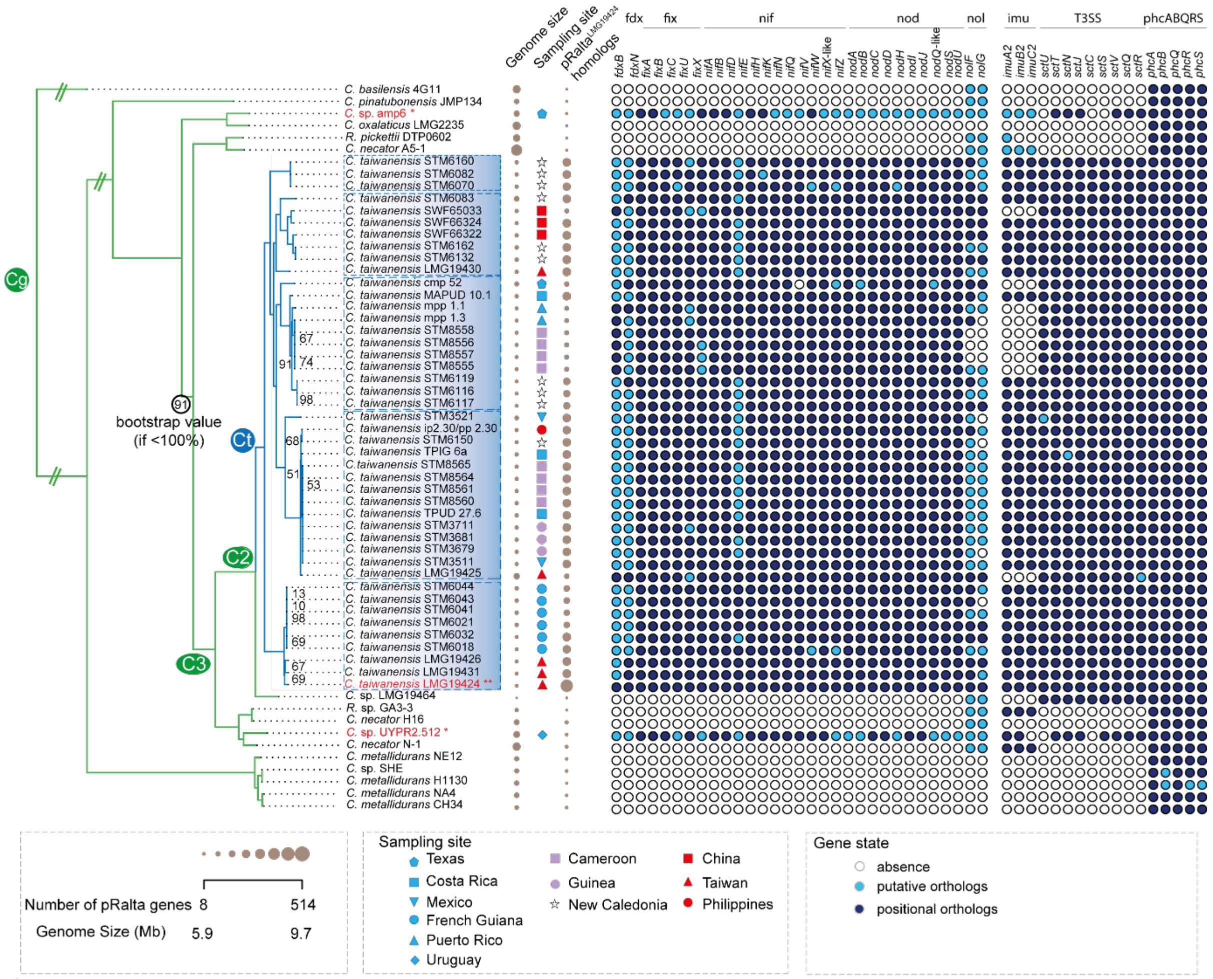

Supplementary Figure 1. Distribution of symbiotic, plasmid *imuA2B2C2*, T3SS and *phcABQRS* genes within *Cupriavidus*. Numbers are ultra-fast bootstrap values (%) reflecting clade support. Nodes with 100% boostrap support (the vast majority) do not have numbers. *: two rhizobia do not belong to *Cupriavidus taiwanensis*. **: reference strain used as pivot to identify orthologs. Clades analyzed in this study are indicated by boxes: Ct, C2, C3 and Cg. White, light blue and dark blue indicate absence, putative orthologs, and positional orthologs, respectively. The size of brown circles for the column "pRalta homologs" is proportional to the number of plasmid genes found in the reference strain *C. taiwanensis* LMG19424. Sampling sites are coded according to geographic origins.

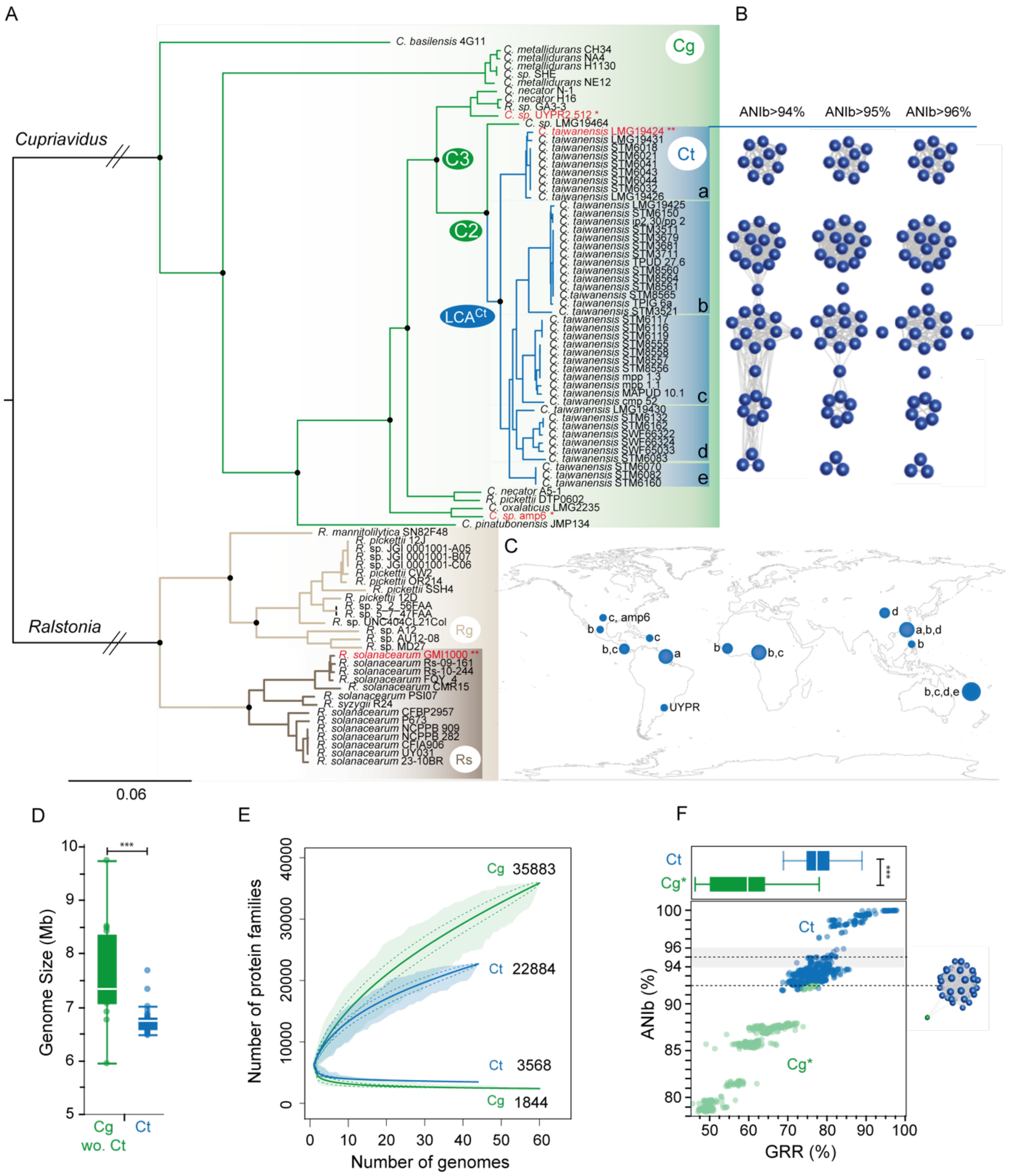

Supplementary Figure 2. Population structure and genomic diversity of *C. taiwanensis*. A. Maximum likelihood phylogenetic tree built using 1003 gene families present in a single copy in all the genomes (see Supplementary Data 2 for taxa and trees with ultra-fast bootstrap values for clade support). The tree was rooted using a 16S rRNA phylogeny with outgroup species (Supplementary Fig. 13). Some strains named *Ralstonia* are actually among *Cupriavidus* (strains GA3-3 and DTP0602). *: two rhizobia do not belong to Ct. **: reference strains used as pivot to compute core genomes. The different clades analyzed in this study using core and pan genomes are indicated by boxes: 2, 3, Ct, Cg, Rs and Rg. The letters (*a* to *e*) in *C. taiwanensis* correspond to putative species. **B**. ANIb cluster analysis of *C. taiwanensis* at different thresholds (see values in Supplementary

Data 4). **C**. Geographic origins of *C. taiwanensis* strains used in this study. The size of the circle indicates the number of strains of each location. The association between the putative species and geographical location was significant ($p<0.0001$, Fisher's exact tests), but that with the plant host species was not ($p=0.11$, same test). **D**. Genome sizes of *C. taiwanensis* and of the rest of *Cupriavidus* strains. **E**. Gene accumulation curves describing the union (pan genome) and intersection (core genome) of gene families obtained by adding a novel genome to a previous set. The procedure was repeated 1,000 times by randomly modifying the order of integration of genomes in the analysis (for lists of core and pan genomes see Supplementary Table 10 and Data 2). **F**. Average nucleotide identity (ANIb) and gene repertoire relatedness (GRR) of the 60 *Cupriavidus* strains. In blue are indicated strains named *C. taiwanensis*, in green other named species. The grey bar around the top dashed line indicates the typical zone of threshold for the definition of a species (94-96% ANIb). The bottom dashed line indicates the maximal ANIb threshold that includes all *C. taiwanensis* in the same species (which leads to the inclusion of strains from other close species). The cluster associated with this threshold is indicated on the right. The differences in terms of GRR between *C. taiwanensis* and the other strains are significant (top boxplots). *** $p < 0.001$, Wilcoxon test.

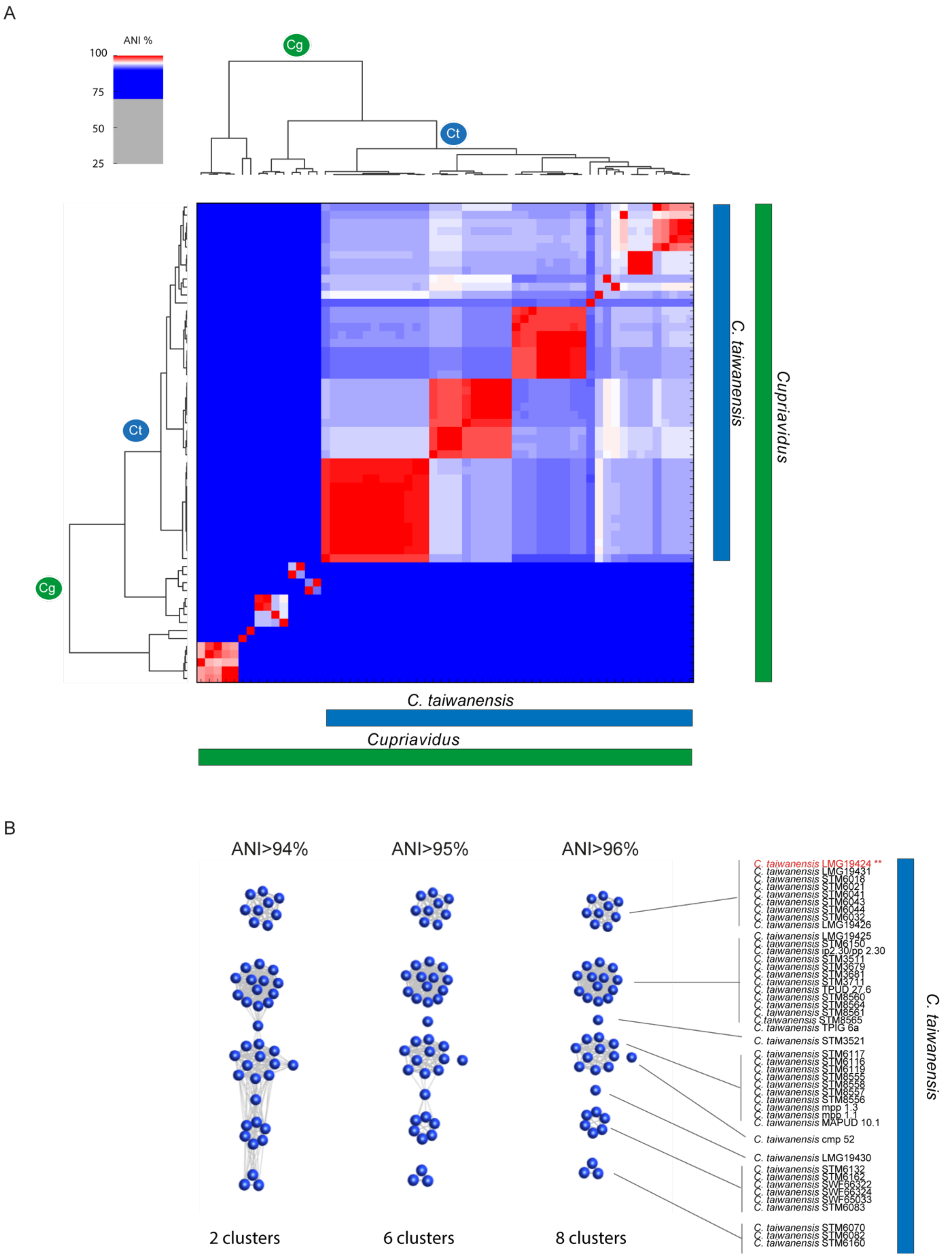

Supplementary Figure 3. Values of ANIb for the 60 *Cupriavidus* strains. A. Heatmap of ANIb values. **B**. Clusters of strains according to ANIb values from 94 to 96%.

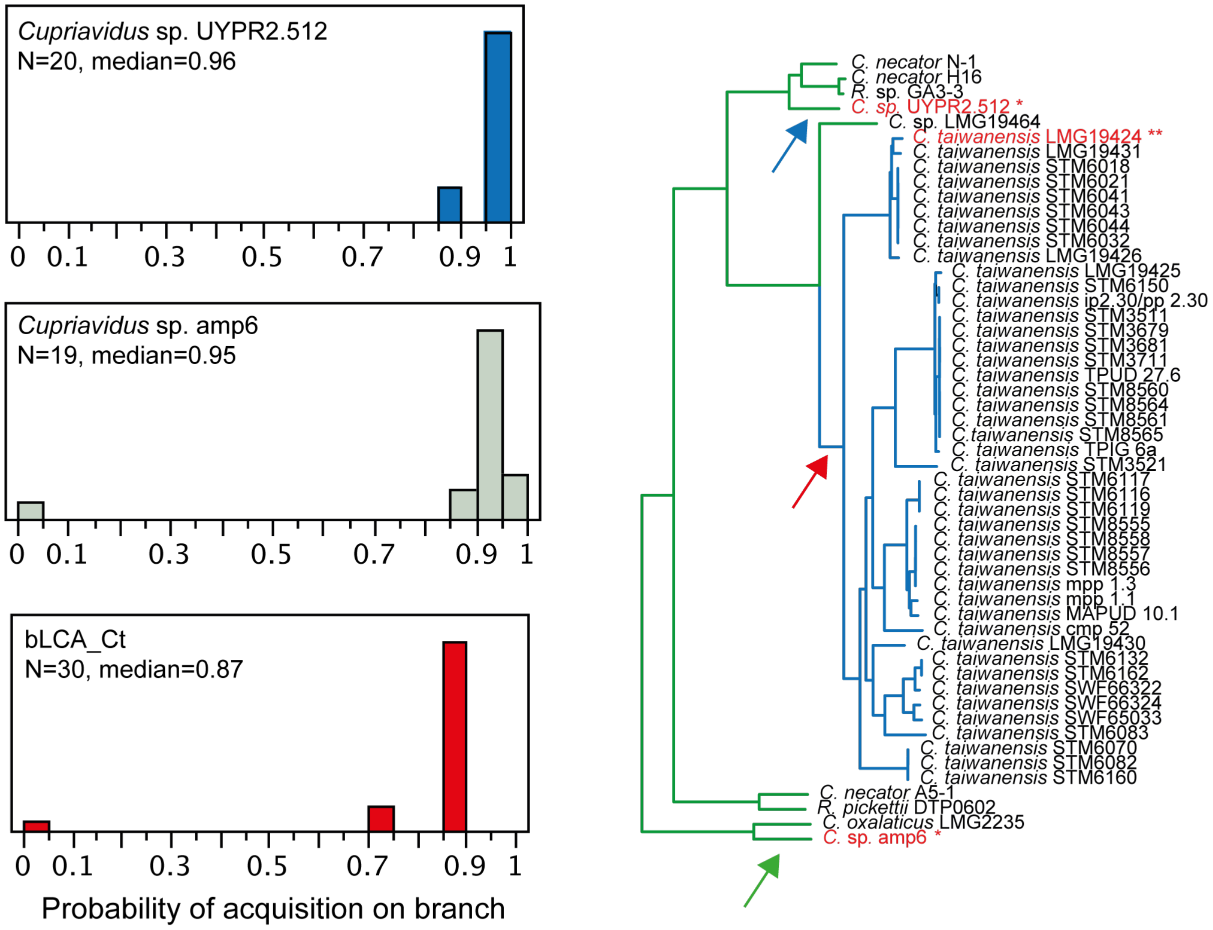

Supplementary Figure 4. The distribution of the probability of the acquisition of *nif-nod* genes in the three branches indicated in the phylogenetic tree. With exception of one gene in two cases (*nolG*), all genes have a probability higher than 50% of being acquired in the branches by HGT.

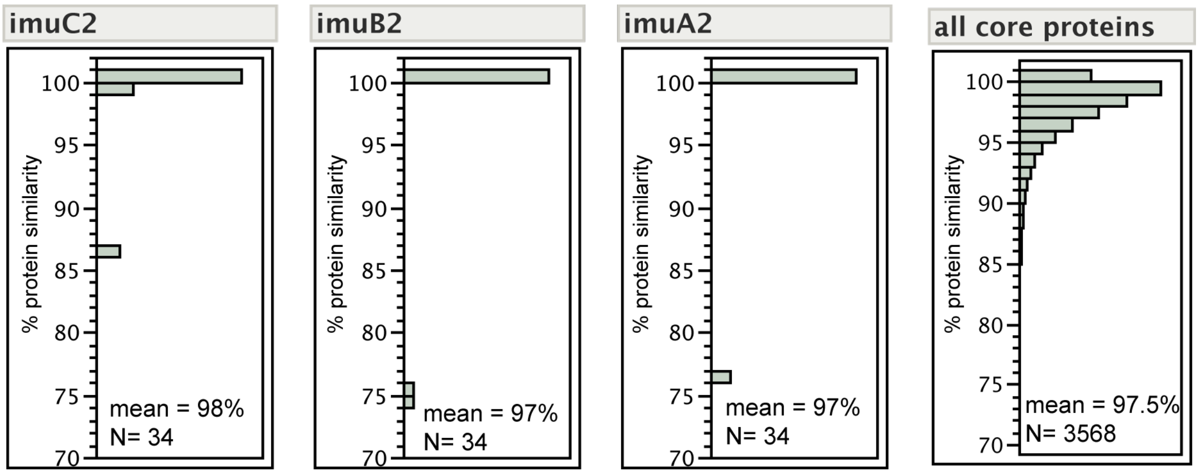

Supplementary Figure 5. Percent similarity between ImuA2B2C2 proteins in *C. taiwanensis* LMG19424 and the remaining *C. taiwanensis* genomes**. On the right, we present the same analysis for all core proteins**. The points below 90% similarity in the first three graphs correspond to the clade STM6041 where (at least in this genome) the locus is carried by another plasmid.

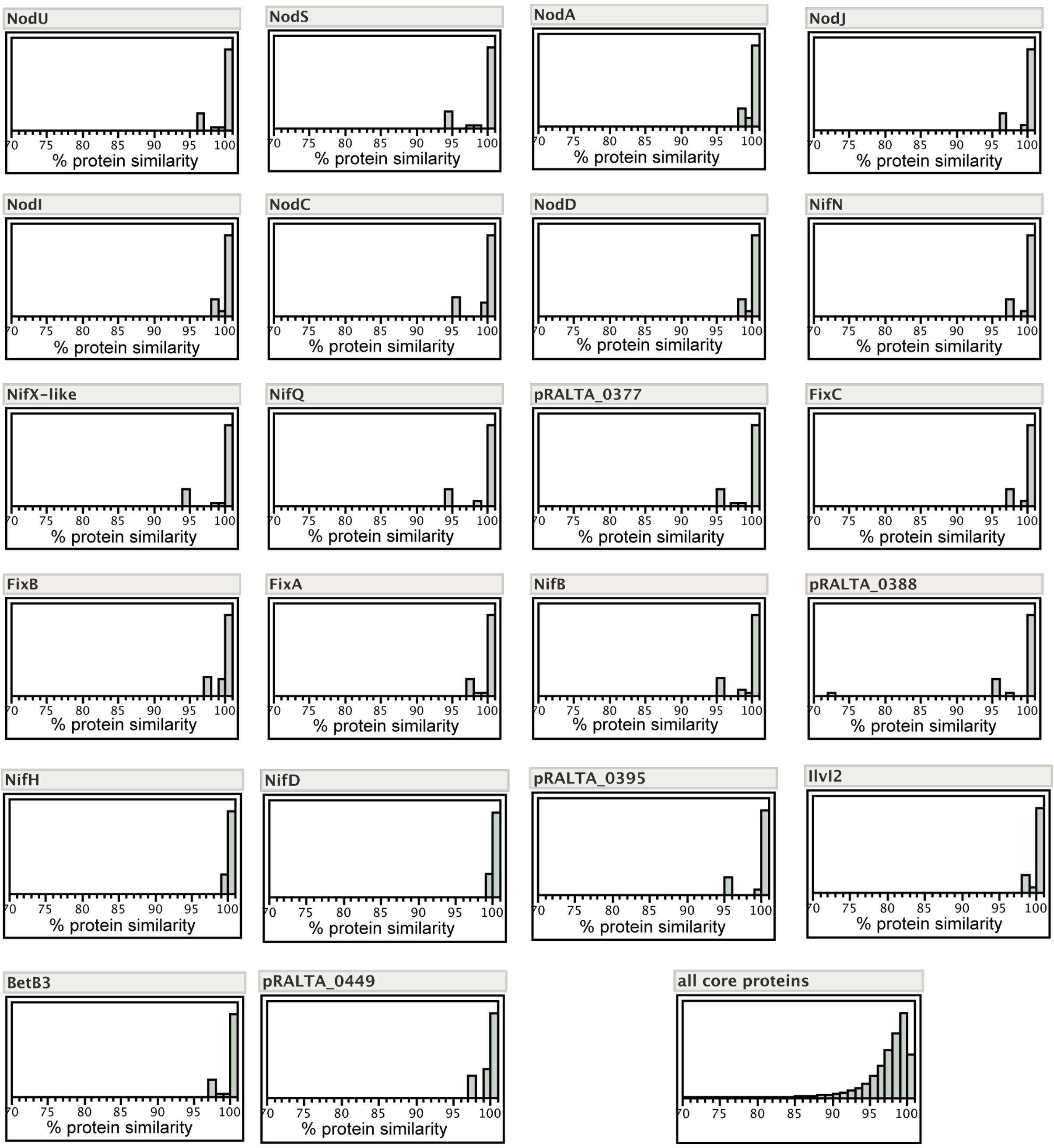

Supplementary Figure 6. Percent similarity between the genes in pRALTA nod-nif locus and similar representation for all core proteins in *C. taiwanensis*. With one single exception, all proteins of the locus had more than 94% similarity with their orthologs in all other genomes of the species.

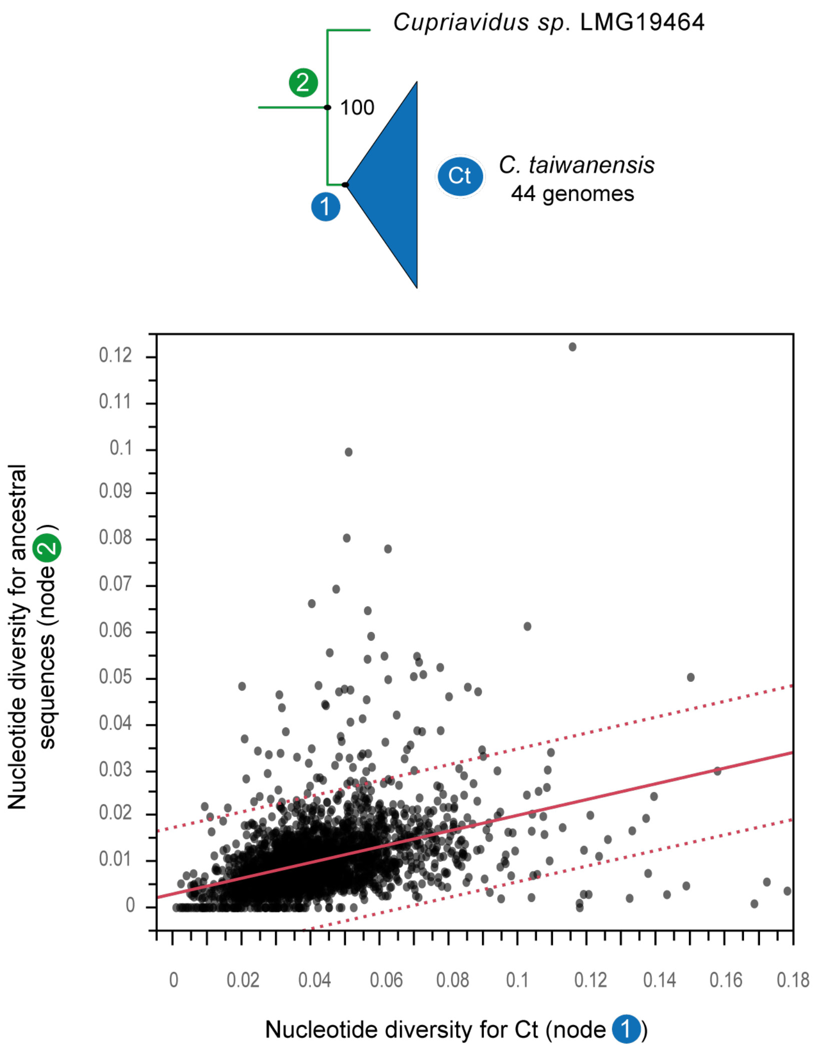

Supplementary Figure 7. Regression analysis between ancestral and ongoing nucleotide diversity of *C. taiwanensis*. Each point represents the nucleotide diversity of a core gene family (core of all genomes under C3 in Fig. 2, X-axis) and the number of genetic differences between LCA^{Ct} and LCA^{C2} (Y-axis). We used a regression analysis to identify genes undergoing rapid genetic diversification in the branch leading to LCA^{Ct} (positive outliers identified using one-sided prediction interval, $p < 0.001$). Dashed lines correspond to the prediction interval (99%). Points above the upper dashed line correspond to positive outliers of LCA^{Ct}, *i.e.*, genes with an excess of diversification.

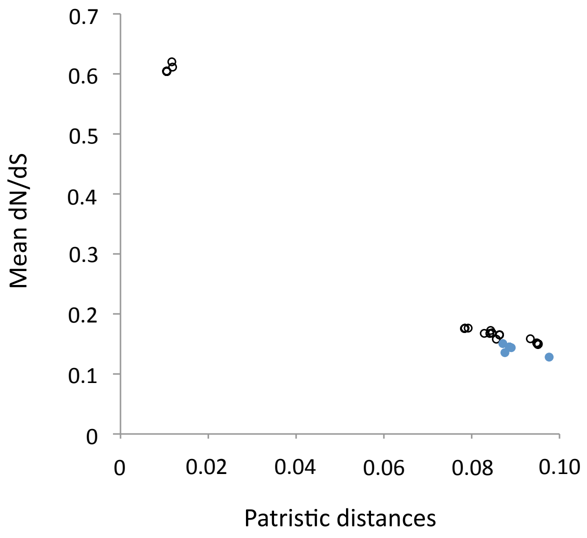

Supplementary Figure 8. Relationship between the average dN/dS and patristic distances for every comparison of *C. taiwanensis* LMG19424 with the other strains. Circles in blue represent strains lacking the *imuA2B2C2* cassette.

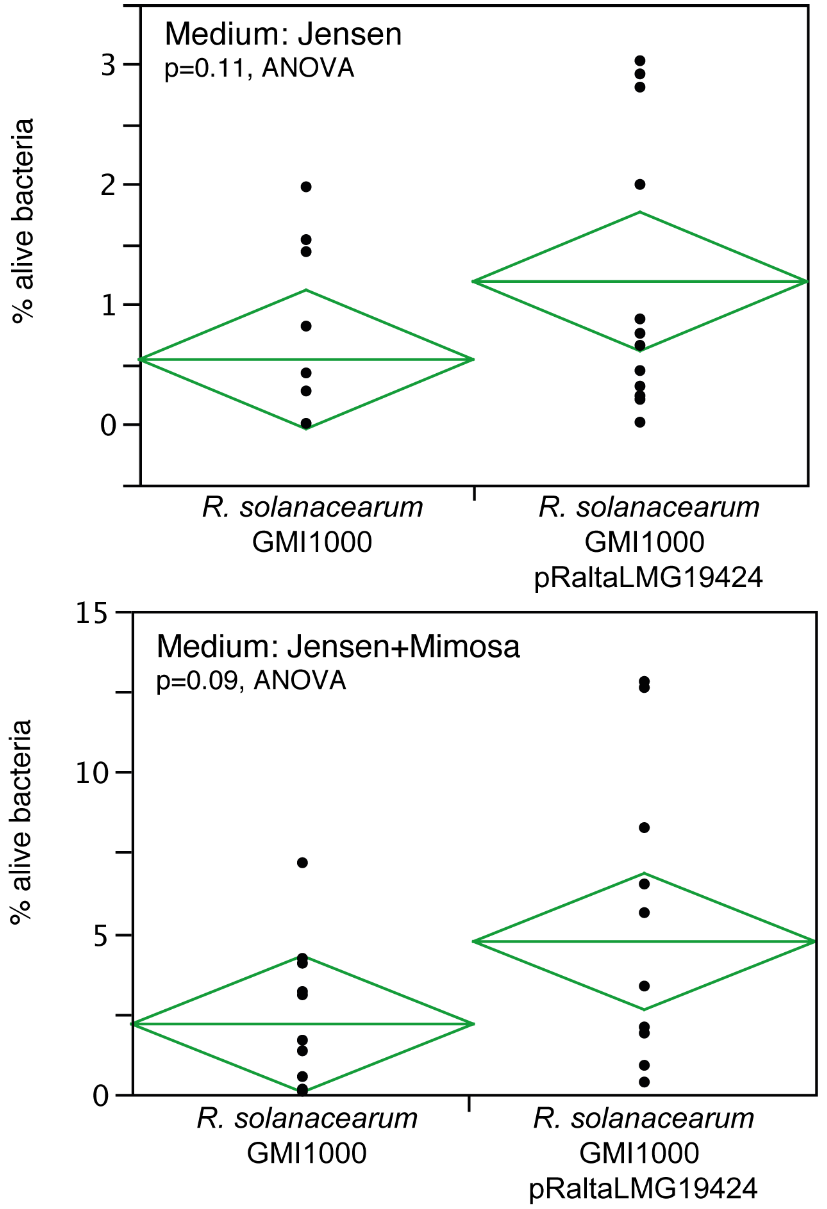

Supplementary Figure 9. Analysis of the cost of the plasmid on original clone. Percentage of alive bacteria cultivated in Gibson tubes filled *with* Jensen medium and containing (bottom) or not containing (top) *Mimosa* plantlets. The values correspond to the last day of the experiment for which all intermediate data is available in Supplementary Table 4 (point at day 22). Each experiment was replicated 12 times. The clone with the plasmid has slightly higher survival rates (indicating a benefit instead of a cost), but the differences are not statistically significant. Means diamonds indicate at the center the mean, and at the edges the 95% confidence interval**.**

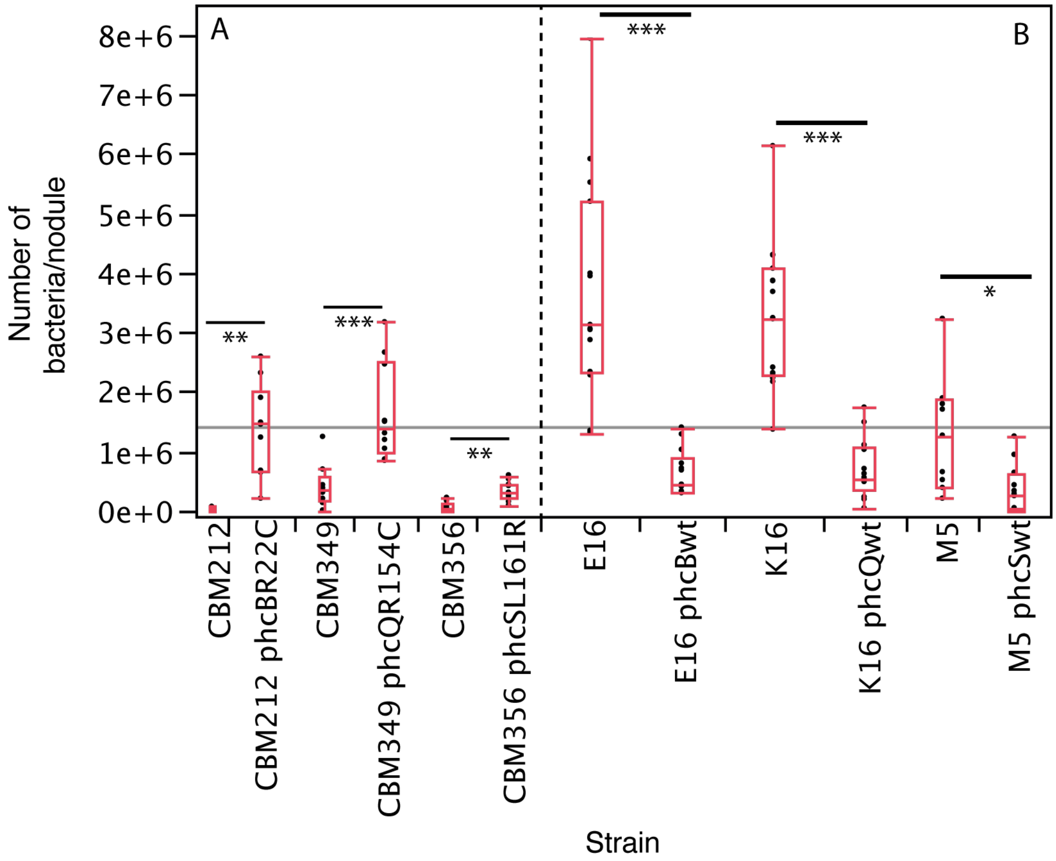

Supplementary Figure 10. Number of bacteria in nodules induced by experimentally evolved clones and *phc* mutant derivatives. **A**. Nodulating ancestors and their mutants containing the mutated *phc* allele. **B**. Evolved clones and their mutants containing the wild-type *phc* allele. Pairwise differences are all statistically significant (Wilcoxon tests): ***$p<0.001$, ** $p<0.01$, * $p<0.05$.

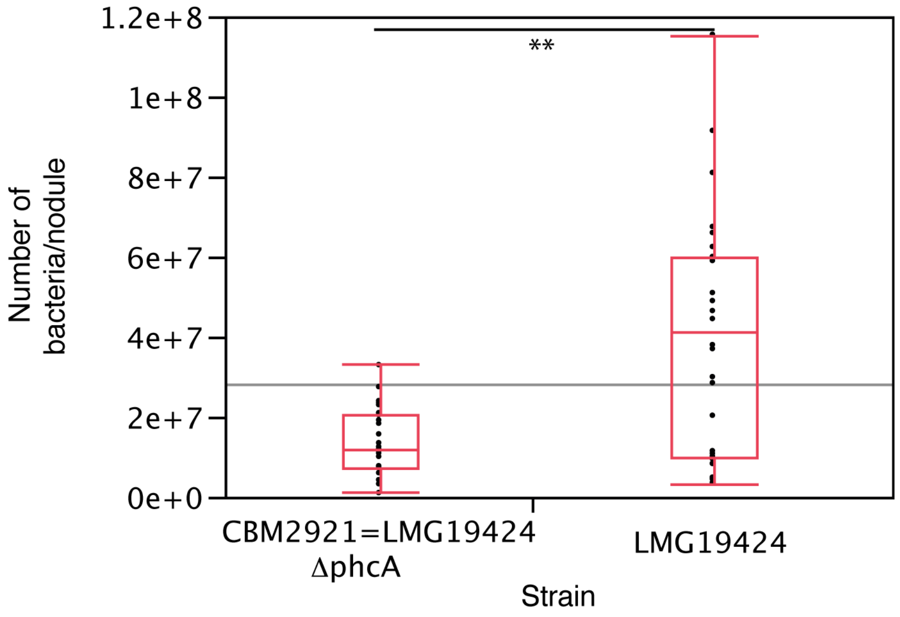

Supplementary Figure 11. Number of bacteria in nodules induced by the *C. taiwanensis* wild-type strain LMG19424 and its *phcA* mutant. Difference is significant (p=0.0014, Wilcoxon test).

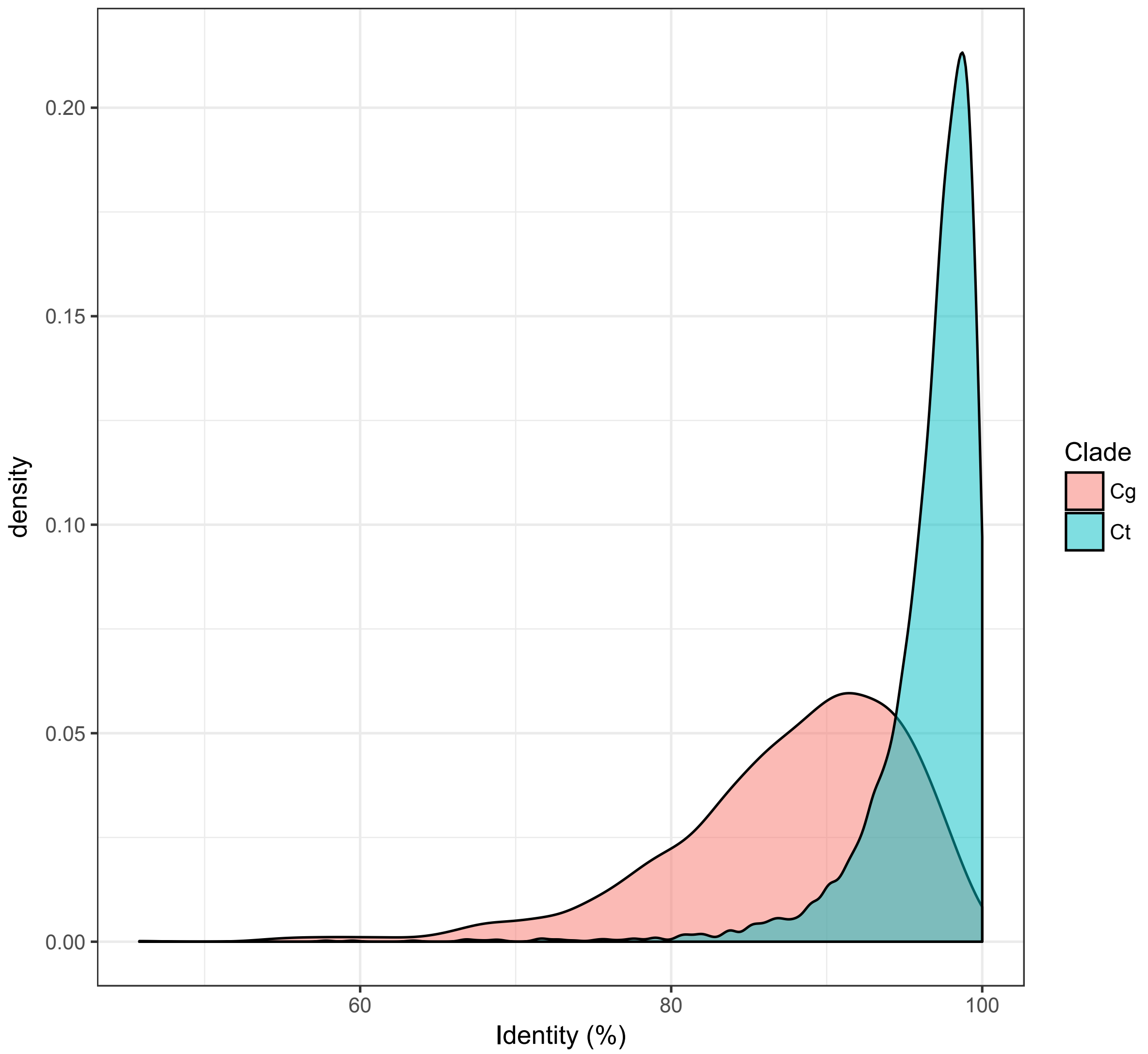

Supplementary Figure 12. Distribution of sequence identity (%) for positional orthologs between: *C. taiwanensis* LMG19424 and *C. taiwanensis* LMG19425 (Ct), and between *C. taiwanensis* LMG19424 and *C. basilensis* 4G11 (Cg).

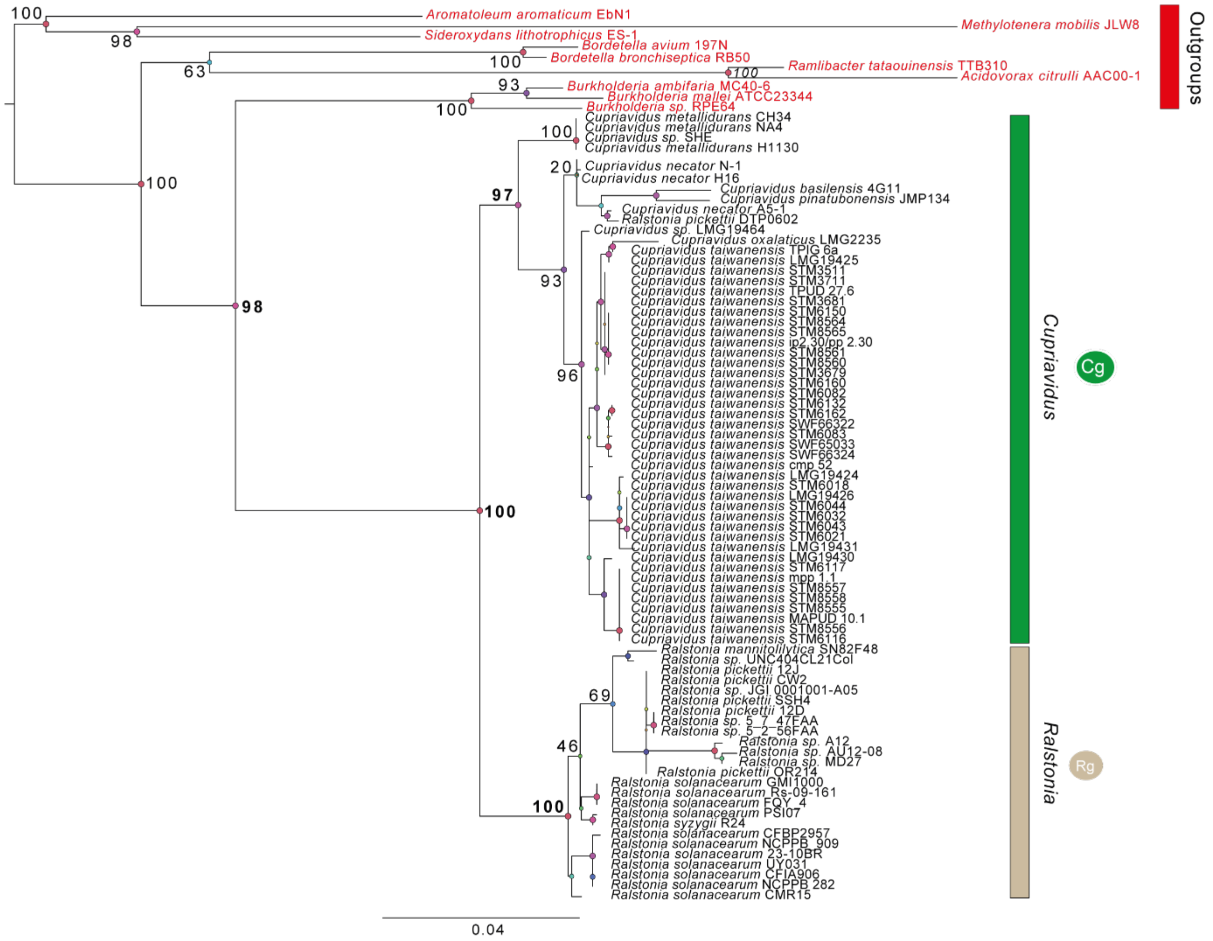

Supplementary Figure 13. Maximum likelihood tree of 16S rDNA sequences. The tree was built using the 16S sequences of *Ralstonia* and *Cupriavidus* strains and 10 outgroup strains to place the root of the core genome tree (Supplementary Fig. 2).

Supplementary Tables

Supplementary Table 1. Large deletions identified in evolved clones. Positions on *R. solanacearum* GMI1000-derived (chromosomes) or *C. taiwanensis* LMG19424-derived (pRalta) replicons are indicated.

Strain	Replicon	Start	End	Length (bp)
**CBM212**	chromosome 2	145944	181821	35877
**CBM212**	pRalta	266782	287131	20349
**CBM349**	chromosome 1	3367183	3396766	29583
**CBM349**	chromosome 2	145944	181821	35877
**CBM356**	chromosome 2	145944	181821	35877
**CBM356**	pRalta	422476	448460	25984
**B16**	pRalta	412675	448108	35433
**C16**	pRalta	412675	448511	35836
**F16**	pRalta	413077	448108	35031
**I16**	pRalta	354052	381753	27701
**I16**	pRalta	434631	458699	24068
**J16**	pRalta	199204	218841	19637
**J16**	pRalta	412969	448217	35248
**K16**	pRalta	416205	448117	31912
**K16**	pRalta	453771	470849	17078
**L16**	pRalta	413041	448146	35105
**N16**	pRalta	357369	400439	43070
**N16**	pRalta	448460	495588	47128
**P16**	pRalta	357175	401208	44033
**P16**	pRalta	448460	495595	47135
**R16**	pRalta	226969	232669	5700
**R16**	pRalta	357254	407448	50194
**R16**	pRalta	448460	485846	37386
**S16**	pRalta	355642	401801	46159
**S16**	pRalta	448460	496958	48498
**T16**	pRalta	357179	400477	43298
**T16**	pRalta	448460	496209	47749

Supplementary Table 2. PacBio dataset.

Strain	Contigs / Unitigs	Reads (#)	Size (bp)	Average Depth*
**STM6041**	1	4995	3387141	35.4
	2	3411	2592224	31.7
	3	822	586737	35.1
	4	473	279233	43.1
	5	319	300529	26.5
**SWF66322**	1	4529	3544531	19.1
	2	2614	2070563	18.4
	3	941	599404	23.6
	4	687	713303	13.9
	5	62	64080	13.1
**STM6160**	1	4610	3638630	30.9
	2	3167	2796790	27.4
	3	766	587959	32.3
**STM3679**	1	6457	3611226	35.5
	2	4550	2792132	32.7
	3	1148	599101	38.6
**STM8555**	1	5412	3563958	35.8
	2	3858	2707856	33.8
	3	815	506723	34.1

* Effective (after assembly)

Supplementary Table 3. Comparisons between natural and experimental evolution. This table indicates the number of genes identified in each type of analysis of the natural evolution and how many genes matched the orthologs with mutations (436 genes) in the experiment. For example: in the natural evolution, we analyzed the excess of diversity (polymorphism) on the branch $bLCA^{Ct}$ using the dataset C3 (see Fig. 2), which has 2849 core genes. Within these genes, 67 had an excess of nucleotide diversity and 27 of them had an ortholog in the chimera used in the experiment. The number of orthologs between the chimera and C3 is 1583 (including only those that could be mapped in the experiment to identify mutations in all clones). Among these genes there were 64 mutations. Seven genes had an excess diversity in the branch $bLCA^{Ct}$ and had a mutation in the identifiable orthologs in the experiment.

			Dataset	Gene families in dataset	# gene positives	Analysed *	# positives w/orthologs	#non-syn w/orthologs	Matches	Comment
Natural evolution	Ancestral events (bLCACt)	Expansions/gains	C3	5887 genes	435	2372	195	151	37	Gains/losses were identified with the pan-genome, enrichment analyses were based on the frequency of functions in LMG 19424
		Positive selection	C3	1676 core genes	46	971	17	35	1	Recombination (PHI) genes removed
		Recombination	C3	2849 core genes	90	1583	46	64	1	-
		Excess of polymorphism LCA^{Ct}	C3	2849 core genes	67	1583	27	64	7	-
	Ongoing events (Ct)	Positive selection	Ct	1869 core genes	325	1012	167	47	6	Recombination (PHI) genes removed
		Recombination (PHI)	Ct	3568 core genes	1699	1773	761	87	40	-
		Recombination (SH)	Ct	3568 core genes	2897	1773	1366	87	64	-
Experimental evolution	Mapped events	Non-synonymous mutations	chimera	5361 genes	436	-	-	-	-	Repeated regions are not mapped in the experiment, coverage corresponds to 88% of the genes

* the analysis of positive selection can only be done in genes lacking evidence of recombination using PHI (hence, the smaller number of genes).

Supplementary Table 4. Genes mutated in the evolution experiment were more likely to be subject to gains or expansions in *C. taiwanensis* and showed an excess of genetic diversity in the branch before LCA^{Ct}. Significant p-values are in bold (Fisher's exact test).

			**Experimental evolution: non-synonymous mutations in mapped genes**	
			**(O-E)/(O+E)**	**P**
Natural evolution	Ancestral events	Expansions/gains	0.62	**1.51E-09**
		Positive selection	0.26	8.01E-01
		Recombination	-0.32	1.00E+00
		Excess of polymorphism LCA^{C1}	0.80	**2.08E-04**
		Nucleotide diversity	0.14	3.37E-01
	Ongoing events	Positive selection	-0.16	8.01E-01
		PHI	0.07	8.01E-01
		SH	-0.10	8.01E-01
		Nucleotide diversity	0.03	3.37E-01

Supplementary Table 5. Observed and expected distribution of synonymous mutations on the three replicons of the final evolved clones. Significant p-values are in bold and correspond to comparisons of observed and expected values using *in silico* genome evolution (see Methods).

Replicon	Observed	Expected	P
Chromosome 1	212	272	1
Chromosome 2	177	145	**0.003**
pRalta	54	24	**0.003**

Supplementary Table 6. Strains and plasmids experimentally used in this study.

Bacterium/plasmid	Strain	Relevant characteristics	Reference
Chimeric *Ralstonia*	RCM1069	GMI1000 IGglmS::SpeR pRalta::Tri, TriR, SpeR	ref [8]
	CBM212	Spontaneous nodulating clone derivative from CBM206, TriR, GenR	ref [8]
	CBM349	Spontaneous nodulating clone derivative from CBM206, TriR, GenR	ref [8]
	CBM356	Spontaneous nodulating clone derivative from CBM206, TriR, GenR	ref [8]
	B16	CBM212-derived evolved clone, cycle 16, TriR, GenR	ref [9]
	E16	CBM212-derived evolved clone, cycle 16, TriR, GenR	ref [9]
	G16	CBM349-derived evolved clone, cycle 16, TriR, GenR	ref [10]
	I16	CBM349-derived evolved clone, cycle 16, TriR, GenR	ref [9]
	K16	CBM349-derived evolved clone, cycle 16, TriR, GenR	ref [9]
	M5	CBM356-derived evolved clone, cycle 5, TriR, GenR	ref [11]
	CBM2034	Evolved clone B16 RSp1236::Spe, TriR, SpeR	ref [10]
	CBM2036	Evolved clone G16 RSp1236::Spe, TriR, SpeR	ref [10]
	CBM2038	Evolved clone I16 RSp1236::Spe, TriR, SpeR	ref [10]
	RCM722 (B16-op)	Evolved clone B16 carrying the original pRalta::Tri, TriR, GenR	this study
	RCM725 (G16-op)	Evolved clone G16 carrying the original pRalta::Tri, TriR, GenR	this study
	RCM806 (I16-op)	Evolved clone I16 carrying the original pRalta::Tri, TriR, GenR	this study
	RCM808 (G16-op)	Evolved clone G16 RSp1236::Spe carrying the original pRalta::Tri, TriR, SpeR	this study
	RCM2288	Evolved clone CBM212 carrying the *phcB*R22C allele and a P*psbA*-GFP fusion downstream *glmS*, TriR, GenR, KanR	this study

	RCM2289	Evolved clone E16 carrying a *phcB* wild-type allele and a P*psbA*-GFP fusion downstream *glmS*, TriR, GenR, KanR	this study
	RCM2290	Evolved clone CBM356 carrying the *phcS*L161R allele and a P*psbA*-GFP fusion downstream *glmS*, TriR, GenR, KanR	this study
	RCM2291	Evolved clone M5 carrying a *phcS* wild-type allele and a P*psbA*-GFP fusion downstream *glmS*, TriR, GenR, KanR	this study
	RCM2292	Evolved clone K16 carrying a *phcQ* wild-type allele and a P*psbA*-GFP fusion downstream *glmS*, TriR, GenR, KanR	this study
	RCM2346	Evolved clone CBM349 carrying the *phcQ*R154C allele and a P*psbA*-mCherry fusion downstream *glmS*, TriR, GenR, KanR	this study
C. taiwanensis	CBM832	LMG19424 derivative resistant to Streptomycin, StrR	M. Hynes
	CBM2921	CBM832 deleted for *phcA*, StrR	this study
R. solanacearum	RCM1068	GMI1000 IGglmS::SpeR	ref [8]
Plasmid	pRalta	Symbiotic plasmid of LMG19424 (0.5 Mb)	ref [12]

StrR, streptomycin resistant. TetR, tetracycline resistant. ChlR, chloramphenicol resistant. TriR, trimethoprim resistant. GenR, gentamicin resistant. GenR, gentamicin resistant. KanR, kanamycin resistant.

Supplementary Table 7. Percentage of alive bacteria *ex planta* in Jensen or Jensen-*Mimosa* medium. The first and second values correspond to survival in Jensen and survival in Jensen-Mimosa, respectively.

		Days post inoculation					
**Strain**	**Replicate**	**0**	**3**	**7**	**10**	**14**	**22**
GMI1000 pRalta^{LMG19424}	1	100/100	57.47/59.79	45.18/40.41	42.03/58.37	16.60/16.48	2.81/1.89
	2	100/100	75.09/56.51	31.33/47.37	7.53/23.88	0.92/21.88	0.76/12.62
	3	100/100	58.59/49.66	7.57/34.77	1.63/26.31	1.42/6.39	0.45/2.08
	4	100/100	43.70/45.94	13.27/33.96	2.87/26.46	1.71/16.72	0.88/5.63
	5	100/100	81.34/48.29	48.92/26.76	19.92/20.58	5.56/8.10	3.03/6.52
	6	100/100	100.00/67.40	45.29/41.61	11.16/29.74	4.32/31.13	2.00/12.81
	7	100/100	84.24/61.88	46.02/52.50	15.06/59.35	5.79/31.6	2.92/8.27
	8	100/100	117.05/44.07	34.01/19.81	23.21/23.13	5.21/19.40	0.02/2.04
	9	100/100	70.34/56.47	44.38/32.59	16.12/19.94	6.03/12.75	0.21/0.89
	10	100/100	82.79/87.50	43.70/54.74	14.96/33.88	9.63/22.54	0.32/3.35
	11	100/100	105.47/48.05	46.01/17.61	14.18/8.74	14.50/3.46	0.66/0.34
	12	100/100	40.60/47.03	1.83/13.44	0.54/3.78	1.47/1.17	0.24/0.37
GMI1000	1	100/100	45.06/48.87	21.61/24.97	9.74/27.38	0.12/2.96	0.01/0.15
	2	100/100	70.72/38.23	48.46/14.62	19.57/1.99	16.27/0.46	1.44/0.50
	3	100/100	81.62/66.43	32.13/48.94	12.46/20.83	3.21/14.20	0.28/4.21
	4	100/100	83.70/94.06	21.07/77.95	3.28/71.66	1.26/35.17	0.43/7.18
	5	100/100	90.40/74.71	54.23/37.75	16.23/22.59	4.17/11.84	1.54/3.08
	6	100/100	100.00/65.93	55.08/42.98	20.61/19.10	6.09/16.61	1.98/4.06
	7	100/100	86.62/67.99	44.40/42.46	18.75/26.70	4.69/13.22	0.82/3.18
	8	100/100	97.24/44.63	33.41/28.08	13.02/21.18	0.01/17.92	0.00/0.54
	9	100/100	73.61/45.35	21.70/18.89	6.37/11.39	0.09/7.90	0.00/0.08
	10	100/100	53.52/51.09	18.58/26.60	7.61/28.60	0.12/19.11	0.01/1.34
	11	100/100	53.27/49.96	10.64/41.91	2.12/29.90	0.09/19.79	0.00/1.67
	12	100/100	56.84/30.48	14.83/12.83	3.75/10.68	0.46/7.14	0.00/0.08

GMI1000 pRalta^{LMG19424} data are from reference [13].

Supplementary Table 8. Primers used to validate large deletions of evolved clones.

Primer pairs					
Forward		Reverse			
Name	Sequence	Name	Sequence	Amplified region	Product length
CBM508	CGCTATGGTGAGGGATCAGT	CBM511	CTCGCCTTCCGAGTAATACG	junction RSp0125-RSp0157	3 kb if deleted
CBM546	GATGCAATCCAAAACCCTGT	CBM547	CGCAGACCAGTTCGTTGTAG	Rsp0138	371 bp if present/0 bp if absent
CBM699	ATGGCACCTCGGTGTTCTAC	CBM700	GTGGCCAGTACCTGACGATT	junction pRALTA_0338 - pRALTA_0311	3.8 kb if deleted
CBM701	CATCCTGGTCATGTTTGTCG	CBM702	ACGGTGCCATCCACTTACTC	pRALTA_0325	554 bp if present/0 bp if absent
CBM703	GCCCAAGACATCTCGTGACT	CBM704	AGAACCGGGAACATCAACAA	pRALTA_0314	426 bp if present/0 bp if absent
CBM719	CCGATCAATCATGCTGACAC	CBM720	TTCGCAAAAATAAGGCGATT	junction Rsc3144 - Rsc3145	1684 bp if present/0 bp if absent
CBM720	TTCGCAAAAATAAGGCGATT	CBM721	TCACTTCGGTCGAACAAGAA	Rsc3122 - Rsc3145	1.9 kb if deleted
CBM721	TCACTTCGGTCGAACAAGAA	CBM722	ATCTGTCGTGCAGGGATCTT	junction Rsc3122 - Rsc3123	1784 bp if present/0 bp if absent
CBM956	CCGGCAAAATAAAAGGTGAA	CBM957	GCTTGTGCCGTCGAAATTAT	pRALTA_0488	419 bp if present/0 bp if absent
CBM958	GATCGCCAATCAGGTTCAGT	CBM959	GAACTTCAGGGTCAGCTTGC	pRALTA_0506	424 bp if present/0 bp if absent
CBM960	ACTGGAGTGGTTCGATACGG	CBM961	TACGAAAAGCAAAGGCGAGT	pRALTA_0431	424 bp if present/0 bp if absent
CBM3129	TACAGCGACTCTATCTGCCG	CBM3130	CGAGACGAGATGGGCATAGT	pRALTA_0240	563 bp if present/0 bp if absent
CBM3131	CAGCTGGGTTAATGCGGATT	CBM3132	CGACAAACTTGGGGATCTGC	pRALTA_0265	388 bp if present/0 bp if absent
CBM3133	AGGCCTATCACACCAACGAA	CBM3134	TGCCAAACGTGTCATTCCAG	pRALTA_0461	633 bp if present/0 bp if absent
CBM3135	GGATCATTCGCCTTGGGTTC	CBM3136	CGAGTGAGACAAGTGGACCT	pRALTA_0471	416 bp if present/0 bp if absent
CBM3137	CGCGATCCAACTACACCAAG	CBM3138	GCTTGTTGTCGGCGCTATTA	pRALTA_0572	487 bp if present/0 bp if absent
CBM3139	GGTGTCGTGCCTGTTTCTTT	CBM3140	ATTTCCTACCCCGGCAAGAT	pRALTA_0593	443 bp if present/0 bp if absent
CBM3141	CGCTTCGATTCAACCGGATT	CBM3142	CAACAGGTGAAAGCGTGACA	pRALTA_0531	486 bp if present/0 bp if absent
CBM3143	CCATGAGCAAGGACAGCATC	CBM3144	AATCCATATTCCCAGGCCGT	pRALTA_0567	403 bp if present/0 bp if absent

Supplementary Table 9. Oligonucleotides used for the construction of mutants.

Primer pairs					
Forward		Reverse			
Name	Sequence	Name	Sequence	Amplified region	Product length
oCBM2652	ACGGTCGAGATGACCAAGAT	oCBM2653	CTGGATCAGCAGGTCCTTGT	amplification of the region around the *phcB*R22C mutation to introduce this mutation into CBM212 or the wild-type allele into E16	6038 bp
oCBM2656	GCCAACTACCAGGGCTATCA	oCBM2657	CCTGCAACACGATGGAAAC	amplification of the region around the *phcQ*R154C mutation to introduce this mutation into CBM349 or the wild-type allele into K16	5982 bp
oCBM2654	ATGCGCTACTTCAGCAACCT	oCBM2655	GTGAGCTGGGACTGGTTGTC	amplification of the region around the *phcS*L161R mutation to introduce this mutation into CBM356 or the wild-type allele into M5	6005 bp
oCBM2673	ACTCCCAGGGTCAGCAGGTGC	oCBM2675	GGCCAGCTCCTGGAACACGT	screening oligos for detecting the wild-type allele of phcB	457 bp
oCBM2674	ACTCCCAGGGTCAGCAGGTGT	oCBM2675	GGCCAGCTCCTGGAACACGT	screening oligos for detecting the mutated allele of *phcB*R22C	457 bp
oCBM3116	GAGCCAGACGCTAACCCAAC	oCBM3118	GTTCGAGATGGTCAAGGGCG	screening oligos for detecting the wild-type allele of phcQ	683 bp
oCBM3117	GAGCCAGACGCTAACCCAAT	oCBM3118	GTTCGAGATGGTCAAGGGCG	screening oligos for detecting the mutated allele of *phcQ*R154C	683 bp
oCBM3157	ACGTTCATCGTGCACTCGGT	oCBM3159	TGTAGCTGGCCGAGACCATG	screening oligos for detecting the wild-type allele of phcS	972 bp
oCBM3158	ACGTTCATCGTGCACTCGGG	oCBM3159	TGTAGCTGGCCGAGACCATG	screening oligos for detecting the mutated allele of *phcS*L161R	972 bp
oCBM3413	GCTCTAGAAGTAACGCGGCAGAGAACG	oCBM3414	CGGGATCCGGTGAGCGAGCAGGACCT	amplification of the phcA upstream region	782 bp
oCBM3415	CGGGATCCGCGGTCATGTGCATCTTCT	oCBM3416	GGAATTCGTGATCCCGCAGCTGAACAA	amplification of the phcA downstream region	812 bp
oCBM3417	GGCGGACGAACGGATAGAAC	oCBM3418	AGGTGCTGAACGACAAGTGG	verification of the phcA deletion (external primer)	2700 bp if wild-typet/ 1882 bp if deleted
oCBM3419	CTGCGCGAAATCTTTGACGA	oCBM3420	GTAGTCGAGCGGGATCAGC	verification of the phcA deletion (internal primer)	528 bp if wild-type / 0 b[illegible] if deleted

Supplementary Table 10. Number of genes and the model used with Count to analyze the pan genomes of *C. taiwanensis* and its genus. Identity (%) indicates the sequence identity cut-off used to compute gene families with SiLiX.

Clade	Number of genomes	Number of families	Identity (%)	Model used in Count
Ct	44	22884	80	-
Cg	60	35883	50	1 category rate variation for loss, length, duplication and gain

Supplementary Table 11. Number of genes identified in the datasets for functional enrichment analyses. This table indicates the references datasets and the associated number of genes used for permutations.

Strain	Natural/ Experimental evolution	Analysis	Number of genes	Reference dataset for comparisons	Number of genes in the reference dataset
C. taiwanensis	Ongoing natural evolution	Positive selection	325	Core-genome of Ct (without genes with significant Phi tests)	1869
		Recombination (PHI)	1699	Core-genome of Ct	3568
		Recombination (SH)	2897		
	Ancestral natural evolution	Expansions/gains	435	Proteome of *Cupriavidus taiwanensis* LMG19424	5887
		Positive selection	46	Core-genome of C3 (without genes with significant Phi tests)	1676
		Recombination	90	Core-genome of C3	2849
		Excess of polymorphism LCA^{Ct}	67		
Ralstonia chimera (*Ralstonia solanacearum* GMI1000 and the symbiotic plasmid of *Cupriavidus taiwanensis* LMG19424)	Experimental evolution	Non-synonymous mutations	436	Mapped proteome of *Ralstonia* chimera	5361

Supplementary References

1. Parker, M. A. A single sym plasmid type predominates across diverse chromosomal lineages of *Cupriavidus* nodule symbionts. *Syst. Appl. Microbiol.* **38,** 417–423 (2015).
2. Richter, M. & Rosselló-Móra, R. Shifting the genomic gold standard for the prokaryotic species definition. *Proc. Natl. Acad. Sci.* **106,** 19126–19131 (2009).
3. Ochman, H. & Moran, N. A. Genes lost and genes found: evolution of bacterial pathogenesis and symbiosis. *Science* **292,** 1096–1099 (2001).
4. Sullivan, J. T., Patrick, H. N., Lowther, W. L., Scott, D. B. & Ronson, C. W. Nodulating strains of *Rhizobium loti* arise through chromosomal symbiotic gene transfer in the environment. *Proc. Natl. Acad. Sci.* **92,** 8985–8989 (1995).
5. Heath, K. D. & Grillo, M. A. Rhizobia: tractable models for bacterial evolutionary ecology. *Environ. Microbiol.* **18,** 4307–4311 (2016).
6. Epstein, B. *et al.* Population genomics of the facultatively mutualistic bacteria *Sinorhizobium meliloti* and *S. medicae*. *PLoS Genet.* **8,** e1002868 (2012).
7. Prior, P. *et al.* Genomic and proteomic evidence supporting the division of the plant pathogen *Ralstonia solanacearum* into three species. *BMC Genomics* **17,** 90 (2016).
8. Marchetti, M. *et al.* Experimental evolution of a plant pathogen into a legume symbiont. *PLoS Biol.* **8,** e1000280 (2010).
9. Marchetti, M. *et al.* Experimental evolution of rhizobia may lead to either extra- or intracellular symbiotic adaptation depending on the selection regime. *Mol. Ecol.* **26,** 1818–1831 (2017).
10. Marchetti, M. *et al.* Shaping bacterial symbiosis with legumes by experimental evolution. *Mol. Plant. Microbe Interact.* **27,** 956–964 (2014).
11. Guan, S. H. *et al.* Experimental evolution of nodule intracellular infection in legume symbionts. *ISME J.* **7,** 1367–1377 (2013).
12. Amadou, C. *et al.* Genome sequence of the -rhizobium *Cupriavidus taiwanensis* and comparative genomics of rhizobia. *Genome Res.* **18,** 1472–1483 (2008).
13. Remigi, P. *et al.* Transient hypermutagenesis accelerates the evolution of legume endosymbionts following horizontal gene transfer. *PLoS Biol* **12,** e1001942 (2014).
