# Supplementary material for: Parallels between experimental and natural evolution of legume symbionts
Source: Nat Commun. 2018 Jun 11;9:2264. doi: 10.1038/s41467-018-04778-5 (PMC5995829; doi:10.1038/s41467-018-04778-5)
Supplement: Supplementary file 3 — Description of Additional Supplementary Files [file 41467_2018_4778_MOESM3_ESM.pdf]

Description of Additional Supplementary Files

File Name: Supplementary Data 1
Description: Mutations observed in the evolution experiments.

File Name: Supplementary Data 2
Description: Core genomes and corresponding phylogenetic trees for the different clades.

File Name: Supplementary Data 3
Description: Information on the genomic datasets of the 89 strains analyzed in this study.

File Name: Supplementary Data 4
Description: ANI and GRR values for the pairwise comparisons among the 60 *Cupriavidus* strains.

File Name: Supplementary Data 5
Description: Functional annotations of *Cupriavidus taiwanensis* LMG19424.

File Name: Supplementary Data 6
Description: Functional enrichment analyses for events that took place in the branch before LCA^{Ct}.

File Name: Supplementary Data 7
Description: Functional annotations of *Ralstonia* chimera and positional orthologs for *Cupriavidus taiwanensis* LMG19424.

File Name: Supplementary Data 8
Description: Functional enrichment analyses for the non-synonymous mutations observed in the *Ralstonia* chimera during the evolution experiment.

File Name: Supplementary Data 9
Description: Functional enrichment analyses for events that took place in the lineage Ct (since LCA^{Ct}).

File Name: Supplementary Data 10
Description: Presence of PhcABQRS genes in *Cupriavidus*, *Ralstonia*, and other genomes of RefSeq.

File Name: Supplementary Data 11
Description: Functional enrichment analyses for events that took place in *R. solanacearum*.
